# A Single, Shared Triploidy in Three Species of Parasitic Nematodes

**DOI:** 10.1534/g3.119.400650

**Published:** 2019-11-06

**Authors:** Ashley Schoonmaker, Yue Hao, David McK. Bird, Gavin C. Conant

**Affiliations:** * Bioinformatics Research Center,; † Department of Plant Pathology,; ‡ Department of Biological Sciences, and; § Program in Genetics, North Carolina State University, Raleigh, NC, 27695

**Keywords:** polyploidy, evolutionary model, root-knot nematode

## Abstract

The root-knot nematodes of the genus *Meloidogyne* are important and damaging parasites capable of infecting most flowering plants. Within this genus, several species of the *Meloidogyne incognita* group show evidence of paleopolyploidy in their genomes. We used our software tool POInT, the Polyploidy Orthology Inference Tool, to phylogenetically model the gene losses that followed that polyploidy. These models, and simulations based on them, show that three of these species (*M. incognita*, *M. arenaria* and *M. javanica*) descend from a single common hybridization event that yielded triplicated genomes with three distinguishable subgenomes. While one of the three subgenomes shows elevated gene loss rates relative to the other two, this subgenome does not show elevated sequence divergence. In all three species, ancestral loci where two of the three gene copies have been lost are less likely to have orthologs in *Caenorhabditis elegans* that are lethal when knocked down than are ancestral loci with surviving duplicate copies.

Root-knot nematodes (Figure 1A) are a group of destructive parasites that attack plant roots and infest a wide variety of crops and other angiosperm lineages across the globe (Trudgill and Blok 2001; Moens *et al.* 2009). Their lifecycle involves the invasion of plant roots, which generally results in the formation of the gall from which the name “root-knot” is derived (Bird 1974). The invasion is followed by the nematodes inducing the plant to form special multinucleated giant cells that nourish the next generation of parasites (Trudgill and Blok 2001). In addition to the direct effects on root performance that may result from infestation (Wesemael *et al.* 2011), the nematodes impose indirect costs on the plant by redirecting a portion of its photosynthetic output to feed the growing nematodes, a cost that can be on the order of 15% of the total energy budget in grape vines (Melakeberhan and Ferris 1989). As a result, the world economic impact of *Meloidogyne* infection is significant, on the order of tens of billions of U.S. dollars annually (Koenning *et al.* 1999). Among the most diverse and damaging of these organisms are the tropical nematodes of the *Meloidogyne incognita* group (MIG), which includes *Meloidogyne incognita*, *Meloidogyne arenaria* and *Meloidogyne javanica* (Trudgill and Blok 2001). These nematodes’ adaptability is illustrated by their rapid invasions of numerous crop species in the few thousand years since the origins of agriculture (Lunt *et al.* 2014). As suggested by the cartoon phylogeny in Figure 1A, the relationships of these three MIG species are still contested, with some published studies proposing that *M. arenaria* and *M. javanica* are sister to each other (Tigano *et al.* 2005; Szitenberg *et al.* 2017) and others arguing for *M. incognita* and *M. arenaria* as sisters (Brito *et al.* 2015; Álvarez-Ortega *et al.* 2019).

**Figure 1:**
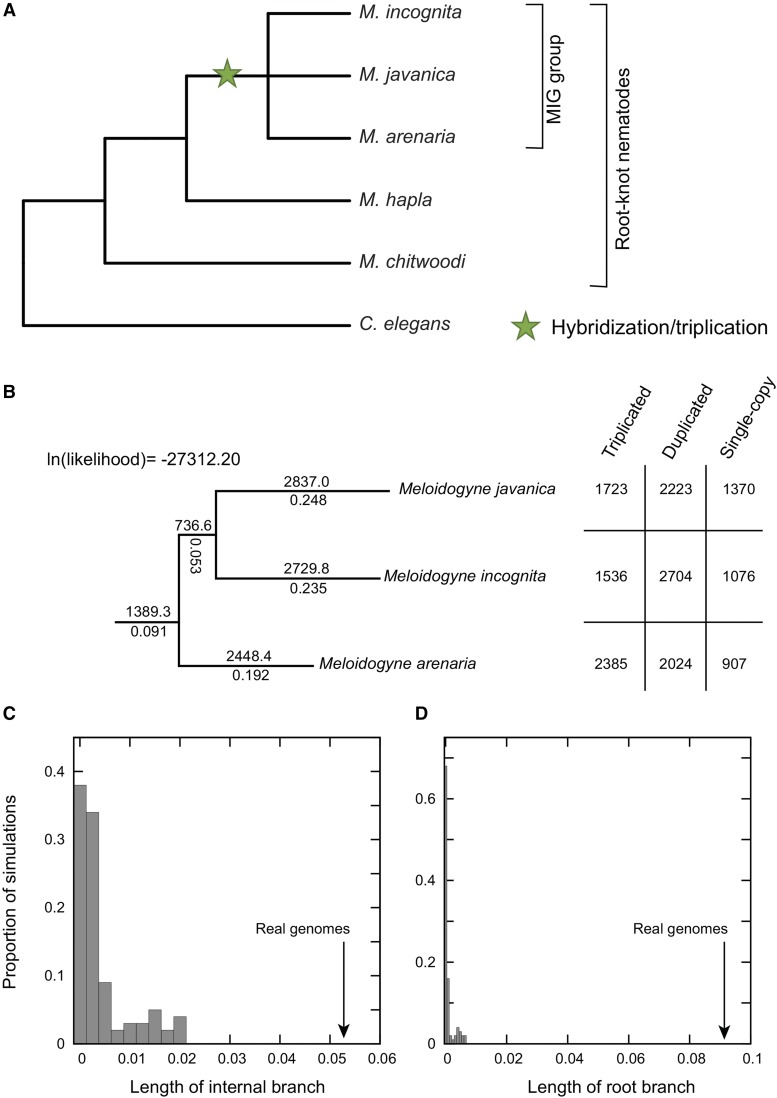
A single, shared triplication inferred across three *Meloidogyne* genomes. A) A schematic phylogeny of the root-knot nematodes showing the placement of the hybridization event shared by the MIG group. The tree topology was adapted from published phylogenetic inferences (Tigano *et al.* 2005; Brito *et al.* 2015; Szitenberg *et al.* 2017; Álvarez-Ortega *et al.* 2019). B) Of the three possible rooted topologies for the three MIG taxa, we show the one that gives the maximum likelihood of observing the set of gene losses found in these genomes (Methods). Branch lengths are reported as the product of the α parameter in Figure 2 and time (αt), while above each branch is POInT’s estimate of the number of gene copies from the triplicated loci lost along that branch. In the table at right are the net number of triplicated, duplicated and single-copy loci in each of three genomes. C) Distribution (*y*-axis) of simulated internal branch lengths (*x*-axis) when the underlying genomes share no common ancestry (see Methods). The actual values from the three genomes correspond to the branch lengths in B and are indicated with arrows for reference. Note that in each simulation, we inferred the maximum likelihood topology for that simulation and use the internal branch from that topology, which may not be identical to the topology in B. D) As for C, but for the root branch.

The genomes of the MIG species contain diverged duplicated gene copies (Szitenberg *et al.* 2017) not present in the diploid genome of the related northern root-knot nematode, *Meloidogyne hapla* (Opperman *et al.* 2008). Although these MIG species diverged from *M. hapla* about 43 MYA, the radiation within the group is likely much more recent, on the order of 5 MYA (Giorgi *et al.* 2002). It now appears that the origins of these MIG species and their radiation was complex: Lunt *et al.* (2014) have proposed that *M. incognita*, *M. arenaria* and *M. javanica* all descend from hypotriplicated hybridization. This hybridization event involved the addition of another copy of the genome to an existing diploid genome, with the subsequent loss of some of the triplicated loci, hence *hypo*triplication (Blanc-Mathieu *et al.* 2017). It was originally suggested that one of the hybrid parents was closely related to the extant *M. floridensis* (Lunt *et al.* 2014), but analyses of new MIG genomes has led to the suggestion that *M. floridensis* is instead a species within the hypotriplicated group (Szitenberg *et al.* 2017). The MIG species’ triploid state has important implications for their biology. In particular it is probably the reason that all are believed to reproduce exclusively by asexual parthenogenesis (Triantaphyllou 1985): indeed polyploidy, hybridization and asexuality appear to co-occur reasonably often, although the causality of this association is imperfectly understood (Neiman *et al.* 2014).

Thus, these MIG genomes can be thought of as originating from a paleopolyploidy event somewhat akin to the paleohexaploidy found in the genus Brassica. In this group of plants, two ancient genomes merged in the first round of hybridization, and a third genome later contributed to form the hexaploid (Tang *et al.* 2012). However, in the nematodes, the process involved one haploid and one diploid gamete, not the merging of two diploid genomes (Lunt *et al.* 2014). It may also be the case that, of the three subgenomes present in these taxa’s genomes, one is more diverged relative to the other two: we will refer to this posited third subgenome as the “diverged subgenome.”

In light of the uncertainties surrounding the evolution of these species, we analyzed three *Meloidogyne* genomes (those of *M. incognita*, *M. arenaria* and *M. javanica*) with POInT, the Polyploidy Orthology Inference Tool (Conant and Wolfe 2008). This tool models the resolution of a polyploidy event along a phylogenetic tree, using gene loss events and shared gene order information (synteny) to infer the combination of phylogenetic relationships, duplicated or triplicated gene loss rates and orthology relationships among the surviving genes that best explains the extant genome structures. As such, POInT is uniquely able to use the patterns of gene loss after the nematode hybridization to provide statistically rigorous answers to four separate questions. First, is there clear evidence for the presence of three distinct subgenomes in these taxa’s genomes? Second, are the three species descended from a single, shared hybridization? Third, are the three inferred subgenomes distinct in their post-hybridization evolution, and can we identify a diverged subgenome? And fourth and finally, does an ancestral gene’s propensity to survive in multiple copies after the hybridization depend on its functional role?

## Methods

### Identifying the relics of an ancestral polyploidy from conserved synteny blocks

We previously created a pipeline for inferring blocks of conserved synteny from a set of polyploid genomes and an outgroup lacking the polyploidy (Emery *et al.* 2018). To understand the polyploid history of the MIG, we applied this pipeline to three nematode genomes, those of *Meloidogyne arenaria*, *Meloidogyne incognita* and *Meloidogyne javanica* (Blanc-Mathieu *et al.* 2017). The genome of *Meloidogyne hapla*, released through Wormbase, was used as the outgroup (Stein *et al.* 2001). This pipeline involves three steps. The first is a homology search of each polyploid genome against the *M. hapla* genome, which was performed with GenomeHistory (Conant and Wagner 2002). Homologous genes between each of the three pairs of genomes were retained if they shared 70% amino acid identity, had a BLAST E-value (Altschul *et al.* 1997) of 10^−9^ or smaller, and had a nonsynonymous divergence (K_a_) less than 0.4.

We used these homologs as inputs to our second step: the search for conserved regions of synteny from the polyploidy. This step places homologous genes in each polyploid genome into blocks of *double* or *triple* conserved synteny (DCS or TCS, respectively): because one of the questions of this analysis was whether this polyploidy was a genome duplication or triplication, we made separate inferences assuming each possibility. This analysis step is based on the concept of a “pillar:” one gene in the *M. hapla* genome and its 1 to 3 homologs in one of the polyploid genomes (*e.g.*, surviving or lost duplicate copies from the polyploidy). Using simulated annealing (Kirkpatrick *et al.* 1983; Conant and Wolfe 2006), we sought a combination of homolog assignments to pillars and a relative order of the pillars themselves that maximized the number of cases where the genes in neighboring pillars were also neighbors in their respective genomes (Emery *et al.* 2018). Finally, in the third step, we merged these inferences across the three polyploid genomes to give a set of pillars with at least one surviving gene in each pillar from each polyploid genome. We then again used simulated annealing to search for a globally optimal pillar order in terms of the number of neighboring pillars that are in synteny in their respective genomes (Emery *et al.* 2018). Under the assumption of a whole genome duplication (WGD), the result was a set of 5,628 pillars with at least one surviving gene from each polyploid genome: the corresponding figure under the assumption of a whole genome triplication (WGT) was 5,316 pillars.

### Modeling evolution after WGD or WGT

**Figure 2:**
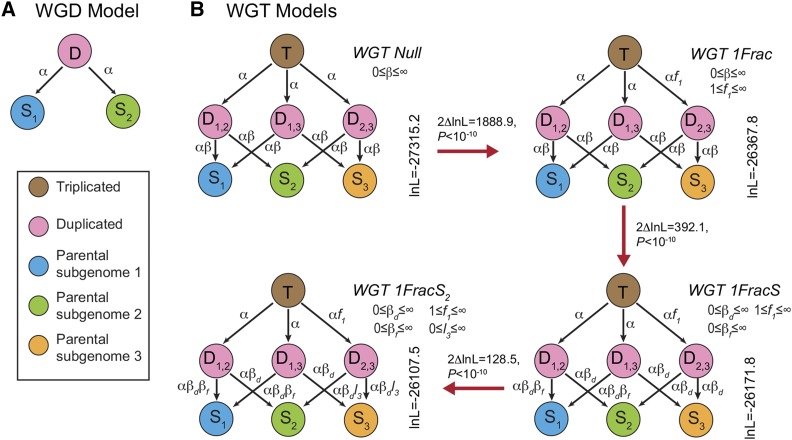
Models of duplicate gene loss after genome duplications or triplications support a biased pattern of gene loss after a genome triplication in members of the MIG group. A) Structure of our null model of evolution after a genome duplication (WGD) and model state definitions. This model was used for the simulation of WGD events for the test of a difference between a WGD and WGT for the three genomes. Duplicated loci from the polyploidy (*D*) can remain duplicated or lose the copy from subgenome 2 (*S_1_*) or subgenome 1 (*S_2_*), losses that occur at instantaneous rate α. B) Nested models of genome triplication (WGT). All loci (pillars) start as triplicated genes (*T*), which may then transition to one of three duplicated states after the first gene loss (*D_1,2_*_,_ *D_1,3_* or *D_2,3_*). Some pillars may experience a further loss from one of the duplicated states, resulting in a single-copy gene at that pillar (*S_1_*, *S_2_*, or *S_3_*), a process that occurs at a rate β relative to the rate of gene losses at triplicated loci. To better understand post-triploidy evolution, we added parameter *f_1_* to the model, allowing the rate of survival of the *S_1_* copy into the duplicated state to differ from the survival rates for genes from subgenomes 2 and 3 (states *S_2_* and *S_3_*, models *WGT Null* *vs.* *WGT 1Frac*). We next allowed for differential loss rates from the duplicated states to *S_1_* verses *S_2_* and *S_3_* (parameters β_d_ and β_f_, model *WGT 1Frac* *vs.* *WGT 1FracS*). Finally, we allowed for differential loss rates to state *S_2_* and to state *S_3_* (parameter *l_3_*, models *WGT 1FracS* *vs.* *WGT 1FracS_2_*). In all three cases, the fit of the model to the observed data, given by the natural log of the likelihood of the genomic data given the tree and model (lnL), improves (see *Results*).

Using POInT (Conant and Wolfe 2008), we fit models of post-WGD and post-WGT evolution to these data (Figure 2). POInT’s computation works in two parts. First, at each pillar, the likelihood of all *2^n^* (for a WGD) or *6^n^* (for a WGT) possible orthology relationships is computed, given the observed gene presence and absence data and one of the evolutionary models shown in Figure 2. In the second step, POInT conditions these *2^n^/6^n^* likelihoods on the corresponding likelihoods for all loci to the left and to the right of the current locus in the ancestral gene order, giving the likelihood of the full set of pillars. The parameters of the model and the tree branch lengths are optimized using standard numerical techniques (Press *et al.* 1992). The resulting optimized likelihood can then be compared to that of other proposed phylogenetic topologies, which, because the models used in POInT are not time reversible (Figure 2), are rooted (Figure 1B). As far as we are aware, there is no other fully integrated statistical modeling tool for studying polyploid genomes that would enable the types of analyses we describe below.

### Comparing the fit of a WGD and a WGT to the MIG genomes

**Figure 3:**
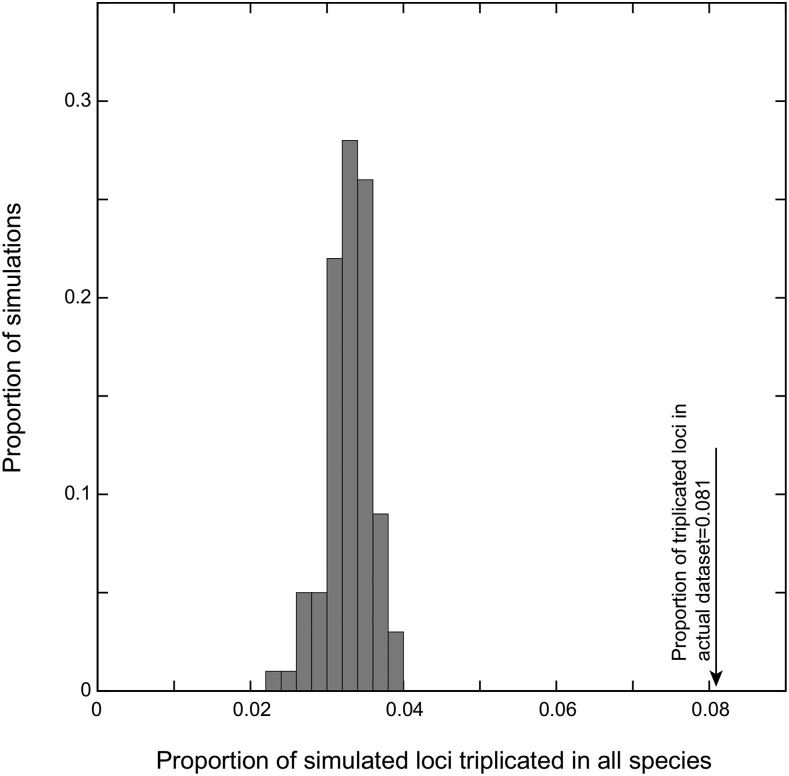
The proportion of loci with three surviving copies of an ancestral gene in all three MIG genomes is greater than expected under a model of genome duplication. On the *x*-axis is the proportion of all loci in the 100 simulated datasets where all three genomes have surviving genes from each of the three subgenomes: on the *y*-axis is proportion of simulations with that value of *x*. The arrow at the right gives this same proportion for the actual MIG genomes: the real genomes possess more shared triplicated genes than can be explained by a genome duplication and chance associations of other homologous genes (see *Methods*).

To test for the presence of a WGT in the MIG, we adopted a simulation approach that sought to assess if the degree of TCS that we inferred in the MIG genomes might be artifactual. To do so, we explored the patterns of TCS that would be observed when the underlying event was known to be a genome *duplication*. We started with our inferred set of optimal DCS blocks from the three MIG genomes. Using those blocks, we simulated 100 WGD events, using the phylogeny of Figure 1B, the WGD null model (Figure 2A) and the maximum likelihood estimates of this model’s parameters. The result of each simulation was a set of single-copy and duplicated loci that follow the original DCS blocks but show different patterns of duplicate loss and retention within them. We then merged the new simulated duplicate genes with all of the remaining homologous genes from each of the three MIG genomes that were not placed into DCS blocks. Thus, each simulation step created three new simulated MIG genomes that mixed simulated single-copy and duplicate genes with these remaining unmapped genes. We then applied our pipeline for TCS block inference to each of these simulated genomes. As a test statistic, we compared the proportion of the total loci from each simulation that were triplicated across all three simulated genomes to this same value from the actual TCS blocks (Figure 3, *Results*).

### Simulation of three independent triploidies

To model the possibility that these three MIG genomes are the products of independent triploidies, we simulated post-polyploidy evolution along a star topology. To do so, we fit the observed pillar data from the three actual MIG genomes to a star topology using POInT (*e.g.*, no shared ancestry between the three genomes). As we have described previously (Conant and Wolfe 2008), we then used POInT to simulate 100 sets of three genomes under this star tree and estimated model parameters. For each of these 100 simulated datasets, we again used POInT to infer the maximum likelihood topology (of the three possible) and extracted the lengths of the root and shared branches. We compared the distribution of the 100 estimates to the length of these two branches seen in the actual dataset, where we had again inferred the maximum likelihood topology (Figure 1B).

### Sequence divergence between parental subgenomes

Using the *WGT 1FracS_2_* model (Figure 2B), we identified surviving triplicated genes from each of the three species where POInT was able to infer the member of the triplet deriving from the derived subgenome with ≥95% confidence. This requirement for high-confidence assignments of genes to the derived subgenome is essential for our analysis but does limit the number of potential triplets for analysis in each MIG genome (see below). For each of the three species, the protein sequences that these triplets of genes code for were aligned with T-Coffee (Notredame *et al.* 2000) and codon-preserving nucleotide alignments inferred. We computed branch-wise estimates of K_s_ and K_a_ for each triplet with our previously described tool (Conant and Wagner 2003). After removing two outlier triplets with abnormal K_s_ values (K_s_ >> 20), 58 *M. arenaria* triplets, 86 *M. incognita* triplets and 34 *M. javanica* triplets were used for further analysis.

Distributions of K_s_ and K_a_ values for genes from different parental subgenomes were visualized using the ggplot2 package (Wickham 2016) in R version 3.4.1. Mann–Whitney *U*-tests (Mann and Whitney 1947) were performed to detect differences between the median of K_s_ for genes from the most fractionated subgenome and that of genes from the two less fractionated subgenomes.

### Identifying M. hapla orthologs of essential and non-essential genes in C. elegans:

Using our published pipeline for using synteny to identify orthologous genes between pairs of genomes (Conant 2009; Bekaert and Conant 2011), we inferred a set of genes from *M. hapla* that are orthologous to genes from the model nematode *Caenorhabditis elegans* (C. Elegans Sequencing Consortium 1998). Briefly, we defined an *M. hapla* gene to be homologous to a gene from *C. elegans* if the two shared a BLASTP (Altschul *et al.* 1997) E-value of 10^−7^ or less and were 30% or more identical at the protein level over 80% of the length of the shorter of the two translated coding regions. From this set of homologs, we defined a pair of genes to be orthologs if either *a*) they were each others’ only homolog in the other genome and had a nonsynonymous divergence (K_a_) less than 0.75 or *b*) they were syntenic neighbors of another pair of orthologs from the two genomes that met criteria *a* or *b* (Conant 2009; Bekaert and Conant 2011). There were 3,162 *M. hapla* genes with identified orthologs in *C. elegans:* of these, 1,988 were among the 5,316 *M. halpa* genes that anchored the pillars of the WGT analysis (see above). We also obtained a list of RNA interference phenotypes for *C. elegans* genes from Wormbase (Howe *et al.* 2017) and extracted from it 3,340 “essential” genes annotated with phenotypes of “embryonic lethal,” “larval lethal,” “adult lethal,” and “lethal.”

### Data availability

Supporting data, including Figure S1, are available through figshare https://doi.org/10.25387/g3.10248929; the POInT software package is available from GitHub (www.github.com/gconant0/POInT).

## Results

### A single, shared, triploidy in the three nematodes

Using POInT, we simulated independent polyploidies for three MIG genomes and estimated the apparent degree of shared ancestry that POInT would infer in the absence of any true shared post-polyploidy ancestry, as we have done previously (Conant and Wolfe 2008). Because we estimated the phylogeny of these three taxa in our analysis of the actual data, we also inferred the optimal topology from each of the simulations and extracted our branch lengths from this optimal tree. As shown in Figure 1C and 1D, the degree of apparent common ancestry seen in polyploidies that are known from the simulation parameters to be independent is much smaller (in terms of phylogenetic branch length) than was the case for our actual dataset (Figures 1C and 1D, *P <* 0.01 in both cases). These results lead us to conclude that these three MIG genomes descend from a single, common hybridization event. Curiously, the maximum likelihood topology inferred with POInT for these three organisms differs from both of the published ones (Tigano *et al.* 2005; Brito *et al.* 2015; Szitenberg *et al.* 2017; Álvarez-Ortega *et al.* 2019): we speculate that it may be difficult to infer phylogenetic relationships among these species with standard gene tree approaches due to the paralogy introduced by the triploidy.

### The three MIG genomes each contain three subgenomes

Formally distinguishing a genome duplication (WGD) from a genome triplication (WGT) is difficult, because a WGD is a special case of a WGT. While the MIG genomes do not exactly fit the common view of polyploids, we can think of the computational problem as that of showing that a WGT better describes the paralogs in these (pseudo)-haploid genomes than does a WGD. We employed a simulation approach that compared the set of TCS blocks that were inferred from a set of simulated genomes known to have undergone only a WGD event to the actual set of TCS blocks from the MIG genomes (*Methods)*. We compared the number of triplicated loci in the simulated TCS blocks to the number seen from the actual MIG genomes. Because we simulated a WGD, not a WGT, any triplicated loci seen in the simulations are due to stochastic effects. The real MIG TCS blocks show many more triplicated loci than the simulated ones, rejecting the null hypothesis that the MIG genomes descend from a WGD event (Figure 3; *P* < 0.01).

### Testing nested models of triplicate loss after WGT

Given the proposed events that generated these three species, we explored a series of nested models describing the evolution of the triplicated loci in the MIG genomes. The simplest model, *WGT Null*, was used for the simulations testing for the presence of the shared WGT above. This model makes the assumption that the rate of loss from all three parental subgenomes is equal (Figure 2B). We then applied a new *WGT 1Frac* model that designates one of the parental subgenomes (corresponding to state *S_1_* in Figure 2) to be less likely to generate surviving members in duplicate states (*D_1,2_*, *D_1,3_* and *D_2,3_*) following the loss of a gene from the triplicated state *T*. The logic of this model is that the hybridization event may have included loci present in the original two subgenomes of the diploid (*S_2_* and *S_3_*), but not in *S_1_*, making states *D_1,2_* and *D_1,3_* disfavored over *D_2,3_*. We then added sequential models that allowed losses from the duplicated states to occur at a different rate to state *S_1_* relative to *S_2_* and *S_3_* (model *WGT 1FracS*) and then also to differ between states *S_2_* and *S_3_* (model *WGT 1FracS_2_*). If we draw a progression of more complex models, from *WGT Null* through *WGT 1FracS_2_*, we find that each prior model is a special case of the new model (Figure 2B). As a result, we can use likelihood ratio tests (Sokal and Rohlf 1995) to assess if the more complex models offer a better fit to our data. Each more complex model in fact offers such an improvement over all its parent models (*P <* 10^−10^ in all cases, Figure 2B). Table 1 gives a breakdown of POInT’s predictions of the number of genes from each subgenome in each of the three MIG genomes.

**Table 1:** Distribution of MIG genes across parental subgenomes

Species	Subgenome 1 (fractionated)*^a^*	Subgenome 2*^b^*	Subgenome 3*^c^*
*Meloidogyne arenaria*	2895.7	4829.1	4385.2
*Meloidogyne incognita*	1980.6	4830.4	4281.1
*Meloidogyne javanica*	2224.7	4769.1	3991.2

a Predicted number of surviving genes (from 5316 total loci) from the most fractionated subgenome (*S_1_* in Figure 2) in each extant genome.

b Predicted number of surviving genes (from 5316 total loci) from the least fractionated subgenome (*S_2_* in Figure 2) in each extant genome.

c Predicted number of surviving genes (from 5316 total loci) from the intermediately fractionated subgenome (*S_3_* in Figure 2) in each extant genome.

### The more fractionated genome does not display elevated sequence divergence relative to the other two subgenomes

**Figure 4:**
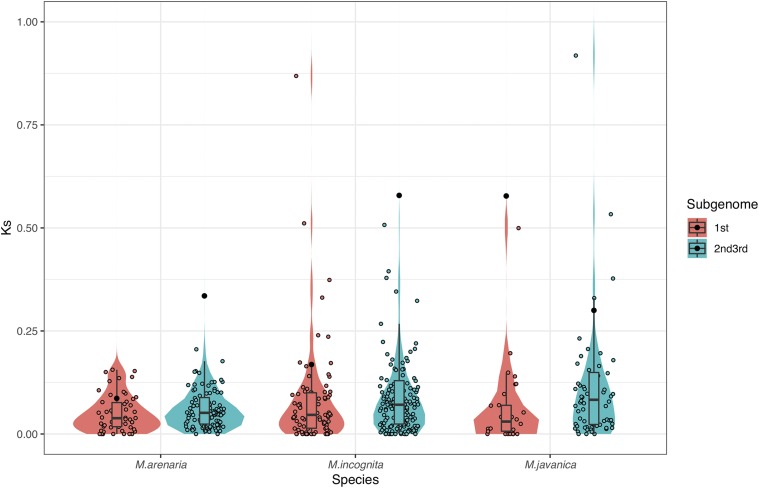
The more fractionated subgenome does not show elevated substitution rates relative to the other two subgenomes. We show the distributions of K_s_ for genes from the more fractionated subgenome (*S_1_* in Figure 2) and the combination of two less fractionated subgenomes. Violin and box plots only show K_s_ values that are smaller than 1, which constitutes more than 90% of the data. Black dots show the mean of all K_s_ values in each group. *P*-values are from one-sided Mann–Whitney *U*-tests of differences between the median of K_s_ values in subgenome 1 and the median of K_s_ values in subgenome 2&3. *M. arenaria P* = 0.04924, *M. incognita P* = 0.01757, *M. javanica P* = 0.00257.

Szitenberg *et al*., (2017) have presented evidence for a mixture of gene copies in the MIG genomes, generally consisting of two very similar copies of each gene, with the addition of a third more diverged copy, and argue for the presence of the hypotriploidy on this basis. Given that our model suggests the presence of a more fractionated subgenome and two less fractioned subgenomes, it was natural to ask if this more fractionated subgenome also showed evidence for greater sequence divergence. However, such was not the case: we analyzed surviving triplicated loci, computing their synonymous and nonsynonymous divergences. We find that, on average, the more fractionated subgenome shows lower branch-specific divergences in K_s_ than do the other two subgenomes (*P =* 0.049, *P =* 0.018 and *P* = 0.003 for *M. arenaria*, *M. incognita* and *M. javanica*, respectively, Figure 4) with little significant difference in K_a_ between the more fractionated subgenome and the other two for two species (*P >* 0.05; *M. arenaria* and *M. javanica*; Figure S1) and a larger mean K_a_ for the more fractionated subgenome for *M. incognita* (*S_1_*; *P* = 0.017).

### MIG genes returned to single-copy are less likely to have essential orthologs in C. elegans

Using our previously described orthology inference tool (Conant 2009; Bekaert and Conant 2011) and data from Wormbase (Howe *et al.* 2017), we compared the proportion of genes in the three MIG genomes that had only a single gene in that pillar (*e.g.*, the other two copies had been lost) that had essential orthologs in *C. elegans* to the same proportion among genes with two or three surviving copies (*Methods*). In all three MIG genomes, the single-copy genes were less likely to have an essential ortholog (*P* ≤ 0.005, chi-square test, Table 2). In *M. incognita*, the surviving triplicated genes were also *more* likely to have essential orthologs (*P* = 0.022), but the enrichment of essential orthologs among the triplicated genes in the other two MIG genomes was non-significant (*P >* 0.05).

**Table 2:** Single-copy genes in the MIG genomes are less likely to have orthologs with knockdown phenotypes in *C. elegans*

Species	Duplicated/Triplicated genes*^a^*		Single-copy genes*^b^*		*P**^c^*
	Lethal knockdown*^d^*	Non-lethal knockdown*^e^*	Lethal knockdown*^d^*	Non-lethal knockdown*^e^*	
*Meloidogyne arenaria*	906	1351	77	183	0.001
*Meloidogyne incognita*	914	1366	69	168	0.001
*Meloidogyne javanica*	833	1231	150	303	0.005

a Genes from *M. hapla* with *C. elegans* orthologs that have an RNA interference phenotype and 2 or 3 surviving homologs from the triploidy in the respective MIG genome.

b Genes from *M. hapla* with *C. elegans* orthologs that have an RNA interference phenotype with only 1 surviving homolog from the triploidy in the respective MIG genome.

c *P*-value of the hypothesis test of equal proportions of lethal knockdown phenotypes in *C. elegans* for the genes with 2 or 3 surviving paralogs and 1 surviving gene: Chi-square test with 1 degree of freedom.

d *M. hapla* genes where the corresponding ortholog in *C. elegans* is lethal when knocked down.

e *M. hapla* genes where the corresponding ortholog in *C. elegans* is not lethal when knocked down.

## Discussion

We confirm two key aspects of the origins of the MIG: namely that they are hybrids with (the remnants of) three subgenomes in each individual and that at least the three species studied descend from a single, common hybridization event. While neither of these findings are likely to be controversial, the modeling framework inherent in POInT allows for testing them in a straightforward and yet rigorous way. Hence, our results tend to further support the hybridization model proposed by Szitenberg *et al*., (2017).

Notably, the three subgenomes found are not interchangeable. As with our previous analyses (Emery *et al.* 2018), we find significant evidence that the three subgenomes differ in their patterns of gene losses: in particular, if we look at the cases were only a single gene of the original three triplicates has been lost, it is much more common for that loss to have been from the fractionated subgenome. This pattern is what would be expected from the proposed hypotriploid model (Szitenberg *et al.* 2017), where the one subgenome either did not possess the gene in question (and so no triplicate was even formed) or would be more likely to be lost due to conflicts with the other two subgenomes. We also observe some evidence for a difference in survival rate between the other two subgenomes (Table 1, *S_2_* and *S_3_* in Figure 2). However, given the somewhat fragmented genome assemblies used, we are reluctant to over-interpret these results, as they may reflect modeling artifacts resulting from the imperfect synteny relationships.

One surprising outcome of these analyses is that while we identified a fractionated subgenome with strong statistical confidence, that subgenome did not show greater sequence divergence from the other two subgenomes (as compared to their own divergences; Figure 4). One could read this finding as contradicting data presented by Szitenberg *et al*., (2017), who suggested the presence of a diverged subgenome based on phylogenetic and sequence identity measures. However, several alternative explanations are possible. Both these and other polyploid genomes show evidence of gene conversion (Evangelisti and Conant 2010; Mcgrath *et al.* 2014; Scienski *et al.* 2015; Szitenberg *et al.* 2017), and it is possible that the diverged gene copies previously detected have moved via gene conversion out of their original syntenic context, making our inferences unable to detect the divergence. A perhaps more likely explanation would be that the more fractionated subgenome is simply not the diverged one: instead there is one distant subgenome that retains many of its genes and two more closely related ones, one of which has lost many genes while the other has not. If the proposal of Lunt *et al.*, that the two similar gene copies are actually also the products of hybridization (Lunt *et al.* 2014), is correct, one could imagine a hybridization of two close relatives, followed by losses in one, followed by the more distant hybridization. This sequence would be consistent with our data and similar to the pattern seen in hexaploid Brassicas (Tang *et al.* 2012). Unfortunately, the fractionation patterns seen are not sufficiently strong for POInT to be able to distinguish loci from subgenomes 2 and 3 with high statistical confidence (data not shown), making it difficult to further analyze any differences in patterns of sequence evolution in these two subgenomes.

These hybrid nematode genomes show patterns of genome evolution that differ significantly from both diploid and other polyploid genomes. The extensive gene loss seen in all three subgenomes (Table 1) is unlike the evolution of diploid genomes. On the other hand, unlike angiosperm polyploids, these three MIG genomes show only a relatively short period of shared evolution by gene loss prior to the speciation event separating *M. arenaria* from the other two species, with only about 1400 gene losses along the root branch (from >5300 *triplicated* loci). In contrast, in angiosperm WGDs, >50% of the duplicated loci had returned to single-copy before the first observed speciation (Emery *et al.* 2018). On the other hand, while the yeast WGD showed few gene losses before the first speciation event (Scannell *et al.* 2007), it was characterized by very even gene loss patterns (Conant 2014; Emery *et al.* 2018), unlike the very definite bias in losses to one subgenome seen here.

The fact that genes returned to single copy in the MIG genomes tend not to have essential *C. elegans* orthologs is in keeping with studies of other polyploid organisms. In yeast and plants, genes that are preserved in multiple copies post-polyploidy tend to be those that were more likely to be essential or show higher selective constraint prior to the polyploidy (Hakes *et al.* 2007; DeLuna *et al.* 2008; Scannell and Wolfe 2008; Hao *et al.* 2018). That a similar (and arguably stronger) pattern is seen here suggests two points: first that conceptually thinking of the MIG nematodes as polyploids can be fruitful, and second that the patterns of which genes did and did not experience gene loss after the MIG hybridization is non-random.

Further study of these nematodes will prove fruitful for a number of reasons. As mentioned, they represent a serious threat to agricultural productivity, and the relationship between their unusual genetics and their propensity to attack crops is still uncertain. There is also a significant body of literature exploring the role played by constraints on relative gene *dosage* in genome evolution (Edger and Pires 2009; Birchler and Veitia 2012), with genes that form part of the same functional complex tending to share the same copy number state after polyploidy (Conant 2014). The fact that the patterns of essentiality seen in the MIG genomes is similar to that from polyploid genomes suggests that similar rules may have governed the loss of genes after this hybridization. However, only an extension of the genome annotations of these organisms can fully validate this hypothesis, because existing data are incomplete. For instance, we investigated whether the previously published Pfam domains found in the *M. hapla* genome (Opperman *et al.* 2008) were predictive of a gene’s propensity to remain in triple-copy after the MIG hybridization, but found no significant effects (data not shown). This null result is likely due to the relatively sparse nature of the functional mapping: no Pfam domain was mapped to more than 94 *M. hapla* genes. With a more refined functional understanding of these genomes, these organisms could be powerful animal models for exploring questions such as the differences between full ploidy changes and aneuploidy, including in human genetic disease (Korbel *et al.* 2009; Pavelka *et al.* 2010; Birchler and Veitia 2012).
